# BAP1 Loss as a Marker of Aggressive Clear Cell Renal Cell Carcinoma: Three Cases With Spinal Metastases

**DOI:** 10.7759/cureus.99724

**Published:** 2025-12-20

**Authors:** Nektarios Alevizopoulos, Dimitrios Alexandris, Vaios Oreopoulos, Georgios N Kanellopoulos

**Affiliations:** 1 Oncology, Evangelismos General Hospital, Athens, GRC; 2 Internal Medicine, Evangelismos General Hospital, Athens, GRC

**Keywords:** axitinib, bap1 mutation, cancer immunotherapy, clear cell renal cell carcinoma, metastatic renal carcinoma

## Abstract

Mutations play a pivotal role in the pathogenesis of renal cell carcinoma. Among these, alterations in the BRCA1-associated protein 1 (BAP1) gene are frequently identified in clear cell renal cell carcinoma (ccRCC) and have emerged as adverse prognostic and predictive biomarkers. We present three cases illustrating BAP1 loss as a key molecular event in a subset of ccRCC associated with aggressive clinical behavior and potential resistance to immunotherapy. Three patients with ccRCC and histologically confirmed thoracic vertebral metastases were retrospectively reviewed. All three demonstrated loss of BAP1 immunostaining and presented with symptomatic spinal involvement at initial diagnosis. None exhibited a meaningful clinical response to localized palliative radiotherapy or to systemic therapy consisting of immunotherapy in combination with axitinib. These observations suggest that BAP1 loss in ccRCC may be associated with early spinal metastatic presentation, reduced survival, and resistance to commonly used therapeutic regimens. Incorporating routine immunohistochemical assessment of BAP1 status into diagnostic evaluation may facilitate earlier identification of aggressive disease and support the development of more individualized treatment strategies aimed at improving clinical outcomes.

## Introduction

BRCA1-associated protein 1 (BAP1) is a tumor-suppressor gene located on human chromosome 3p21.3 and encodes a ubiquitin carboxy-terminal hydrolase [1]. It is considered a prototypical “two-hit” tumor-suppressor gene [1]. Within the nucleus, BAP1 acts as a chromatin scaffold that facilitates chromatin-remodeling complexes, thereby regulating cell proliferation through the deubiquitylation of host cell factor 1 (HCF1) [2]. In the cytoplasm, BAP1 localizes to the endoplasmic reticulum, where it stabilizes the type 3 inositol-1,4,5-trisphosphate receptor (IP3R3). This stabilization promotes calcium-mediated cytochrome c release from mitochondria, ultimately triggering apoptosis [3]. In addition, BAP1 has been implicated in broader cellular metabolic processes [4]. For example, one study compared plasma samples from 16 BAP1+/- individuals across two families carrying diverse germline BAP1 mutations with samples from 30 BAP1-wild-type controls from the same pedigrees [4].

Germline BAP1 mutations have also been identified in individuals with familial mesothelioma and familial melanocytic tumors [5,6]. A comprehensive meta-analysis of all published BAP1-mutated families demonstrated strong associations between BAP1 mutations and malignant mesothelioma, uveal melanoma, and cutaneous melanoma, thereby defining a distinct hereditary cancer syndrome now referred to as the BAP1 cancer syndrome [7,8].

In clear cell renal cell carcinoma (ccRCC), BAP1 mutations are observed in approximately 14% of cases [1]. Several studies have linked BAP1 loss to higher-grade tumors, and a molecular subtype of ccRCC defined by concurrent VHL and BAP1 mutations has been proposed [9]. Since this initial observation, numerous investigations have examined the prognostic significance of BAP1 loss in ccRCC and its potential impact on response to therapeutic interventions [1].

## Case presentation

**Figure 1:**
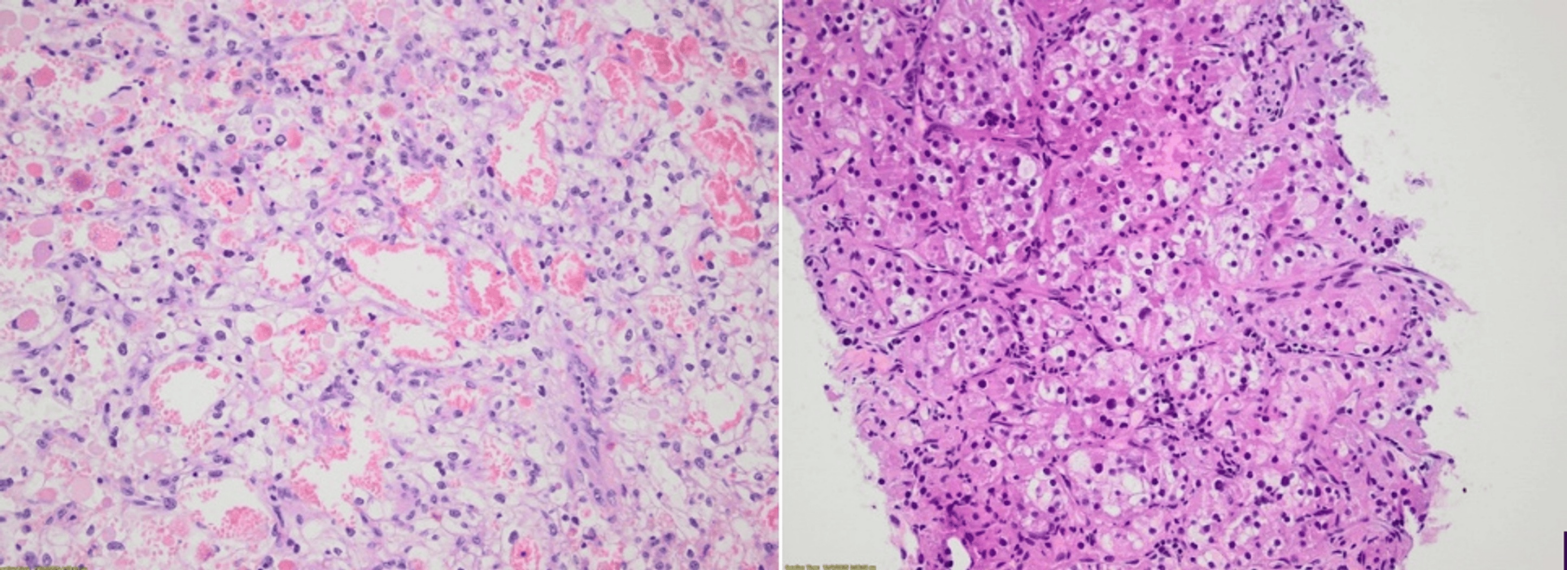
Nests and tubules of polygonal cells with distinct cell borders and optically clear cytoplasm, containing irregular hyperchromatic nuclei, some with prominent eosinophilic nucleoli, are observed in association with a rich arborizing capillary network in two patients (hematoxylin and eosin stain).

**Figure 2:**
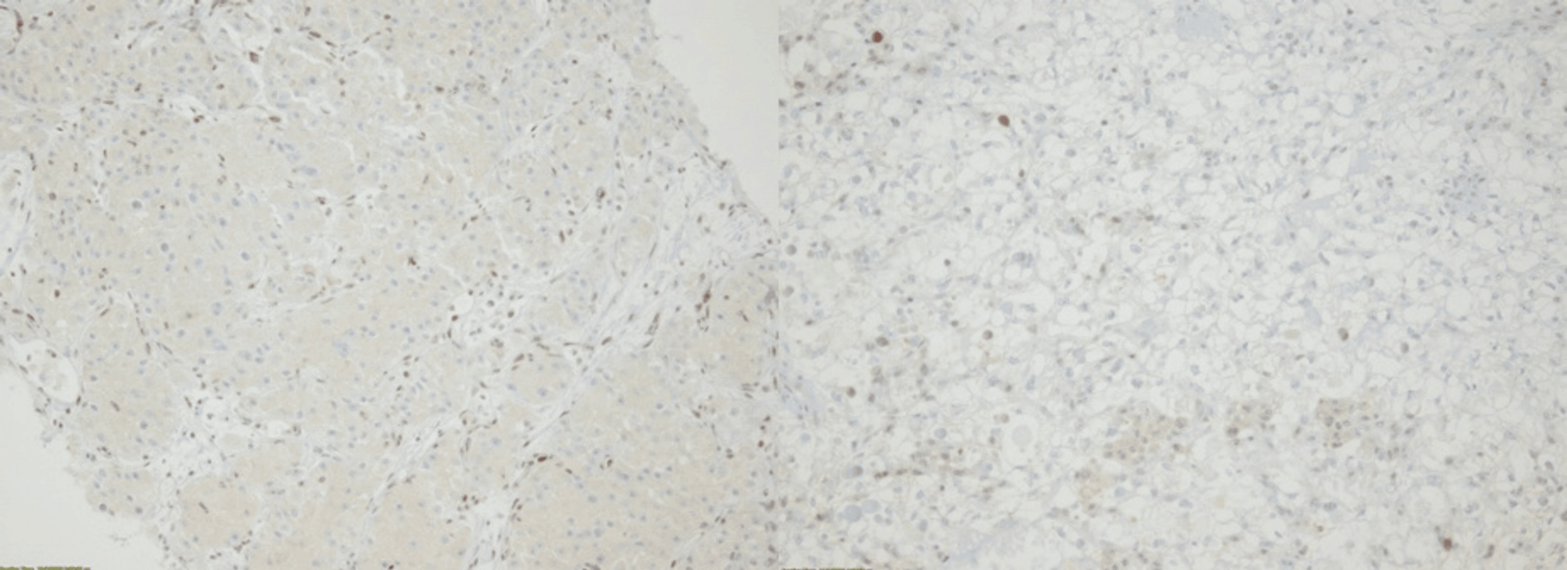
Immunohistochemical labeling for BAP1 protein in patients with clear cell renal cell carcinoma, showing loss of nuclear staining. Immunostain: BAP1.

Herein, we present three cases of patients with extensive metastatic involvement of the thoracic vertebrae secondary to confirmed ccRCC. All tumors demonstrated complete loss of BAP1 immunostaining, a finding consistently associated with poorer prognosis and adverse clinical outcomes (Figures 1, 2). In all cases, spinal involvement was the initial clinical presentation, with thoracic pain serving as the predominant symptom that ultimately prompted further evaluation and led to the diagnosis.

**Figure 3:**
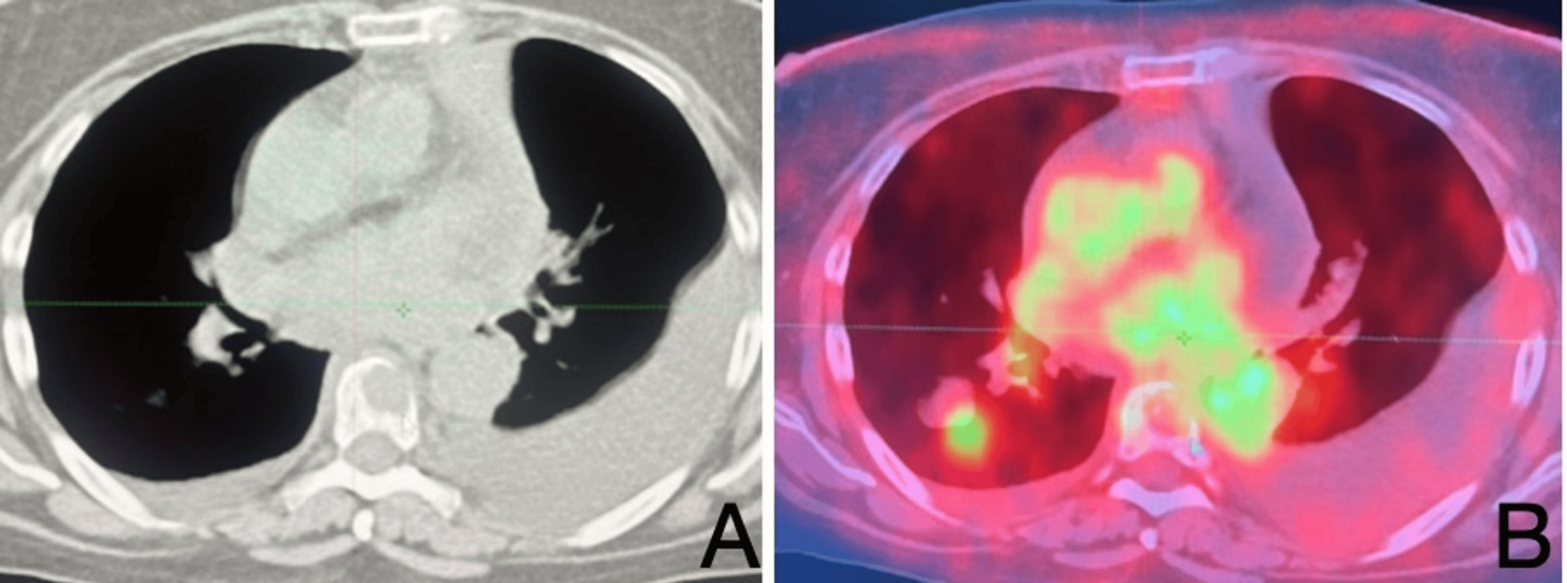
Patient 1: (A) CT scan showing a malignant mass infiltrating the thoracic vertebral body. (B) PET-CT demonstrates intense hypermetabolic activity in the same lesion, consistent with metastatic thoracic spine involvement. CT: computed tomography; PET-CT: positron emission tomography-computed tomography.

**Figure 4:**
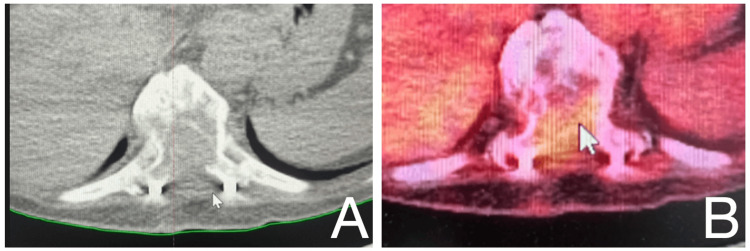
Patient 2: (A) CT scan showing destructive malignant infiltration of the thoracic vertebral body. (B) PET-CT demonstrating marked hypermetabolic activity corresponding to the same vertebral lesion, consistent with metastatic involvement. CT: computed tomography; PET-CT: positron emission tomography-computed tomography.

**Figure 5:**
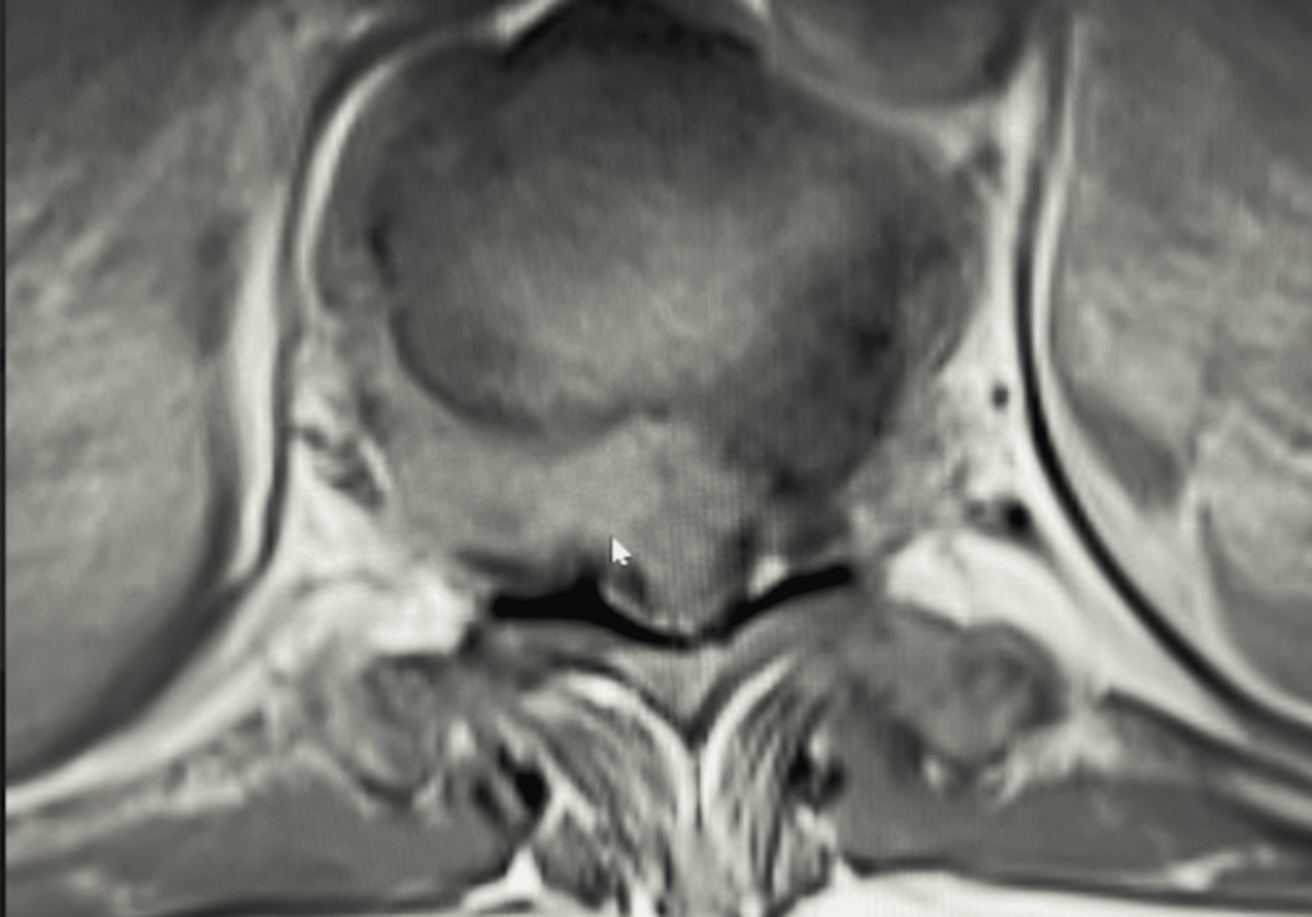
Patient 3: MRI demonstrating a mass within the thoracic cavity encroaching upon the bone-localized marrow, consistent with metastatic infiltration of the thoracic vertebral structures.

In case 1, a male patient was found to have a malignant mass infiltrating the thoracolumbar region (Figure 3). Case 2 involved a female patient with a malignant neoplasm encroaching upon the thoracic spine, as demonstrated on CT and PET-CT imaging (Figure 4). In case 3, a male patient presented with a thoracic cavity mass extending into the bone marrow compartment (Figure 5).

Comprehensive imaging in all three patients identified an underlying renal mass, prompting fine-needle aspiration of the affected lesions. Histopathological evaluation confirmed ccRCC in all cases, with subsequent immunohistochemical analysis demonstrating complete loss of BAP1 expression.

All patients received palliative radiotherapy for symptom control; however, none demonstrated a satisfactory clinical response to local treatment. Consequently, each patient was initiated on combination systemic therapy consisting of immunotherapy with pembrolizumab (200 mg intravenously every 21 days) and the tyrosine kinase inhibitor axitinib, in accordance with current first-line therapeutic recommendations for metastatic renal cancer.

Regrettably, all three patients experienced immune-related adverse events, including colitis and thyroid dysfunction, which were manageable; however, none achieved a durable clinical response to first-line therapy. Their recorded progression-free intervals were only two, three, and three months, respectively, with minimal disease stabilization in each case. All patients were subsequently transitioned to second-line therapy with everolimus, which likewise failed to produce a meaningful therapeutic response.

Further molecular and cytogenetic analyses are currently underway to better characterize their tumor profiles and to elucidate potential mechanisms underlying their uniformly poor outcomes. These cases underscore the clinical challenge posed by renal cell carcinoma with BAP1 loss at initial histological assessment. The lack of responsiveness to conventional systemic therapies highlights an urgent need to investigate alternative, more precisely targeted treatment strategies for this distinct patient subset.

Importantly, clarifying the biological mechanisms of resistance associated with absent BAP1 expression, an aberration increasingly linked to aggressive tumor behavior in renal cell carcinoma, may prove crucial in guiding future therapeutic approaches. The observed correlation between BAP1 loss and the adverse clinical trajectories in these cases warrants further investigation and may ultimately inform the development of more effective treatment paradigms.

## Discussion

Across the majority of published studies, BAP1 alterations have been associated with significantly poorer prognosis compared to patients without such changes [1]. Tumors demonstrating both immunohistochemical loss and genetic mutations of BAP1 generally indicate worse outcomes regardless of treatment modality, although some inconsistencies remain between studies [1]. Prior investigations have also suggested that BAP1 mutations may correlate with biomarkers of responsiveness to immune checkpoint inhibitors [1]. Notably, BAP1 loss is more frequently observed in sarcomatoid and rhabdoid tumors, subtypes known to respond better to immunotherapy [1]. Other studies have linked BAP1 loss to a more inflammatory tumor microenvironment, which could support improved immunotherapeutic response [10].

In our patient cohort, however, we did not observe such favorable responses to immune checkpoint inhibitors, despite the expected poor prognosis associated with BAP1 alterations. This finding underscores the complex and not fully understood relationship between BAP1 status and treatment outcomes [1]. The mechanisms driving these divergent immunotherapy responses require further clarification, particularly as BAP1 mutations continue to emerge as clinically meaningful prognostic biomarkers in several malignancies [1,11].

Although current literature generally reports that BAP1 mutations are associated with unfavorable outcomes, our observations highlight the need for a more detailed understanding of how these alterations influence disease behavior [1]. Integrating BAP1 mutation status, protein loss, and immune-related characteristics may help refine treatment selection and improve clinical decision-making within the setting of targeted and immune-based therapies.

Future research should aim to identify the key molecular pathways affected by BAP1 loss, which may reveal new therapeutic opportunities. A better understanding of the biological mechanisms underlying BAP1-associated tumors could improve prognostic accuracy and support the development of more personalized treatment strategies.

Interdisciplinary collaboration among oncologists, pathologists, and molecular researchers will be essential to advance knowledge in this area. Comprehensive molecular studies will be needed to clarify the mechanisms of carcinogenesis and identify predictive biomarkers that can guide individualized treatment for metastatic ccRCC.

The cases presented in this report highlight the complexity of managing advanced ccRCC, especially when extensive metastatic disease and BAP1 alterations are present. Continued investigation into the therapeutic implications of BAP1 loss may provide valuable insight that ultimately improves patient outcomes in the era of precision medicine.

## Conclusions

The immunohistochemical detection of BAP1 loss in ccRCC has been associated with reduced survival and a higher incidence of adverse clinical outcomes. This association extends beyond poor prognosis to include suboptimal responses to established therapeutic approaches, such as long-standing anti-vascular endothelial growth factor agents and newer immune checkpoint inhibitor combinations. Recognizing this pattern may increase clinician awareness of early metastatic progression in renal cancer and inform the development of novel therapeutic strategies with improved potential for clinical effectiveness.
